# Correction to Effect of Vitamin D Supplementation on Circulating Level of Autophagosome Protein LC3A, Inflammation, and Physical Performance in Knee Osteoarthritis

**DOI:** 10.1111/cts.70324

**Published:** 2025-08-11

Saengsiwaritt W, Jittikoon J, Chaikledkaew U, Tawonsawatruk T, Honsawek S, Udomsinprasert W, “Effect of Vitamin D Supplementation on Circulating Level of Autophagosome Protein LC3A, Inflammation, and Physical Performance in Knee Osteoarthritis,” Clinical and Translational Science, 2023 16(12):2543–2556. doi: 10.1111/cts.13646.

In the funding information section, the funding sources were incorrectly stated as:

“This work was supported by the National Research Council of Thailand (NRCT; N42A650217) and the Royal Golden Jubilee (RGJ) PhD Programme Year 2020 (NRCT5‐RGJ63012‐145).”

The correct acknowledgment should read:

“This research project is supported by National Research Council of Thailand (NRCT): NRCT5‐RGJ63012‐145 and Grant N42A650217.”

We apologize for this error.

